# Technology Readiness Level and Self-Reported Health in Recipients of an Implantable Cardioverter Defibrillator: Cross-Sectional Study

**DOI:** 10.2196/58219

**Published:** 2025-02-06

**Authors:** Natasha Rosenmeier, David Busk, Camilla Dichman, Kim Mechta Nielsen, Lars Kayser, Mette Kirstine Wagner

**Affiliations:** 1 Department of Public Health University of Copenhagen Copenhagen Denmark; 2 Department of Cardiology Copenhagen University Hospital Rigshospitalet Copenhagen Denmark

**Keywords:** implantable cardioverter defibrillator, health literacy, self-management, ICD rehabilitation, digital health literacy, patient-reported outcome measure, self-reported, self-rated, exploratory, interview, sociodemographic, survey, cluster analysis, mixed method, cross-sectional, Denmark

## Abstract

**Background:**

Approximately 200,000 implantable cardioverter defibrillators (ICDs) are implanted annually worldwide, with around 20% of recipients experiencing significant psychological distress. Despite this, there are no ICD guidelines addressing mental health as part of rehabilitation programs, which primarily focus on educating patients about their condition and prognosis. There is a need to include elements such as emotional distress, social interactions, and the future use of technologies like apps and virtual communication in ICD rehabilitation, without increasing the burden on health care professionals.

**Objective:**

This study aimed to demonstrate how data from the Readiness for Health Technology Index (READHY), combined with sociodemographic characteristics and exploratory interviews, can be used to construct profiles of recipients of an ICD, describing their ability to manage their condition, their need for support, and their digital health literacy. This aims to enhance health care professionals’ understanding of different patient archetypes, serving as guidance in delivering personalized services tailored to the needs, resources, and capabilities of individual recipients of ICDs.

**Methods:**

Overall, 79 recipients of an ICD participated in a survey assessing technology readiness using the READHY. The survey also collected sociodemographic data such as age, sex, and educational level. Self-reported health was measured using a Likert scale. Cluster analysis categorized participants into profiles based on their READHY scores. Correlations between READHY scores and self-reported health were examined. In addition, qualitative interviews with representatives from different readiness profiles provided deeper insights.

**Results:**

Four technology readiness profiles were found: (1) profile 1 (low digital health literacy, insufficient on 5 dimensions), (2) profile 2 (sufficient on all dimensions), (3) profile 3 (consistently sufficient readiness on all dimensions), and (4) profile 4 (insufficient readiness on 9 dimensions). Participants in profile 4, characterized by the lowest readiness levels, were significantly younger (*P*=.03) and had lower self-reported health (*P*<.001) than those in profile 3. A correlation analysis revealed that higher READHY scores were associated with better self-reported health across all dimensions. Qualitative interviews highlighted differences in self-management approaches and the experience of support between profiles, emphasizing the essential role of social support toward the rehabilitation journeys of recipients of an ICD. Two patient vignettes were created based on the characteristics from the highest and lowest profiles.

**Conclusions:**

Using the READHY instrument to create patient profiles demonstrates how it can be used to make health care professionals aware of specific needs within the group of recipients of an ICD.

## Introduction

Worldwide, approximately 200,000 implantable cardioverter defibrillators (ICDs) for primary and secondary prophylactic indications are implanted every year [1]. In Denmark, 2000 people were treated with an ICD in 2020 [2]. It is evident that implantation of an ICD with a primary prophylactic indication significantly improves the survival of patients with high-risk cardiovascular conditions who have symptomatic heart failure and a left ejection fraction below 35% [3]. Despite a significant benefit on reduction in mortality in recipients of an ICD [4] and the fact that most recipients effectively adapt to life with an ICD [5], a systematic review involving 45 studies and over 5000 recipients found that approximately 20% of recipients of an ICD experience clinically significant psychological distress [6]. Despite the acknowledged issue, there are currently no national or international ICD guidelines that specifically address the management of mental health issues as an integral component of rehabilitation. Previously, it has been proposed that rehabilitation programs should incorporate customized, hospital-based services tailored to the unique requirements and preferences of recipients of an ICD, with the aim of ensuring adequate psychological well-being and overall quality of life [5,7]. Currently, the initial rehabilitation program after discharge comprises activities aimed at enhancing understanding of the underlying disease and prognosis, as well as preparing the recipient for life with an ICD. However, there is a need to incorporate specific elements addressing the individual’s unique challenges, such as emotional distress, perceived lack of support, or other person-specific concerns [8]. This necessitates the development of innovative approaches in clinical care and rehabilitation without increasing the demand for additional hours from health care professionals. A study involving individuals with chronic obstructive pulmonary disease [9] recommends incorporating both virtual and in-person components to enhance adherence [10]. To obtain the benefits of this approach, we suggest implementing similar strategies in ICD rehabilitation, as shown to be beneficial in the chronic obstructive pulmonary disease study.

When proposing the use of digital services and technology, it should be noted that approximately one-third of the older adult population in Denmark lacks a sufficient level of health literacy or digital health literacy [11]. It may be assumed that a significant number of recipients of an ICD are also challenged if expected to actively engage with digital health information. This number may even increase if the recipients are expected to participate in web-based activities in relation to a rehabilitation program. However, the challenge may be greater for recipients of an ICD than for other groups with long-term health conditions, as many recipients of an ICD are burdened by cognitive impairment as a consequence of a recent cardiac arrest, heart failure, general arteriosclerotic disease, or psychological distress [12,13]. We consider it essential, in the design of a new rehabilitation program, to address the individual needs of recipients of an ICD in relation to the heterogeneity of this group, with respect to their ability to manage their condition, their need for support, and their digital competencies. Such a redesign will enhance both the patient experience and assist in a more efficient allocation of health care professional’s resources. This may involve providing virtual or even generative artificial intelligence–based services to individuals who are digitally literate and allocating in-person hours to those who require more personal contact due to social exclusion. Based on previous research involving patients with inflammatory bowel disease [14], patients with type 2 diabetes mellitus [15], and cancer survivors [16], we hypothesize that by using a patient-reported outcome dataset, such as the Readiness and Enablement Index of Health Technology (READHY) [16], alongside supplementary data on sociodemographic characteristics, it is feasible to map individuals’ perceived support, self-management capabilities, and digital health literacy. This approach can facilitate the creation of patient profiles, thereby enhancing health care professionals’ awareness of the diverse needs of their patients.

The READHY is a validated instrument that consists of 13 dimensions with a total of 65 items related to self-management, social support, and digital health literacy. The instrument builds on the concept of digital health literacy as the core measured with the validated eHealth Literacy Questionnaire (eHLQ; 7 dimensions), supplemented with 4 dimensions reporting on aspects of self-management from the Health Education Impact Questionnaire (heiQ) and 2 dimensions reporting on support from the Health Literacy Questionnaire (HLQ) [17-19].

The purpose of this study is to demonstrate, in the context of recipients of an ICD, how READHY data, supplemented with sociodemographical characteristics and explorative interviews, can be used to create profiles of recipients of an ICD, describing their needs, resources, and capabilities with respect to their technology readiness.

## Methods

### Study Design

The study consisted of a mixed methods, cross-sectional design in 2 parts; part one encompassed a quantitative analysis, while part two involved a qualitative inquiry. In the first part, the analysis of READHY data led to the creation of 4 profiles based on participants’ self-management capabilities, perceived support levels, and digital health literacy (technology readiness). Subsequently, individuals representing high and low levels of technology readiness were invited for interviews. This approach was used to provide a voice to these profiles and to illustrate the varying perspectives within the group of recipients of an ICD.

### Setting, Recruitment, and Participants

Participants included in this study were recipients of an ICD who participated in the voluntary ICD rehabilitation meeting following implantation at the Department of Cardiology at the University Hospital of Copenhagen, Rigshospitalet. The ICD rehabilitation meetings were conducted on a monthly basis, and each recipient attended only once after their device implantation. The purpose of the meeting was to address common questions about living with an ICD; provide general information and guidance about the technology behind the ICD; and explore how the treatment affects both the patient and their close relatives, including both physical and mental health issues. The meetings were facilitated only in person and by specially trained nurses, physiotherapists, and ICD technicians from the Department of Cardiology. Eligible participants were adults with primary and secondary prophylactic indications. During the research period, a total of 743 ICD devices were implanted. All patients received verbal information about the voluntary ICD rehabilitation meetings before discharge. At their first post-ICD visit, they were provided with a written invitation to the available meetings. A total of 82 (11%) patients out of 743 attended the meetings, where all completed the READHY assessment. Of these, 3 were excluded: one received a pacemaker instead of an ICD, one did not complete all of the READHY assessment, and one attended the meeting twice. The meetings were not formal hospital appointments but were offered as an additional resource for patients seeking further support and information. The inclusion took place from November 2019 to May 2022. In November 2021, a total of 6 participants, selected from a pool of 38 individuals, were invited to take part in individual semistructured interviews. In total, 3 recipients were identified from a profile of 26 individuals characterized by high levels of technology readiness, while the other 3 recipients were identified from a profile of 12 individuals with particularly low levels of technology readiness. The selection and invitation of participants was facilitated by the author, MKW, among those still in an active follow-up program at Rigshospitalet.

### Sociodemographic and Technology Readiness

A survey consisting of the READHY, sociodemographic characteristics, and self-reported health were administered at the meetings [19]) consist of between 4 and 6 items, which all have a 4-point response scale ranging from “strongly disagree” to “strongly agree.” An average score ranging from 1 (strongly disagree) to 4 (strongly agree) was calculated for each of the dimensions. The heiQ8 “emotional distress” dimension is reversed by subtracting the scores from a value of 5 for the purpose of analysis, as normally a high score would mean a high level of distress. The reversed scale now means a high level of distress has the lowest score equal to 1, so a higher score means less emotional distress as reported in the validation of the instrument [16].

**Figure 1:**
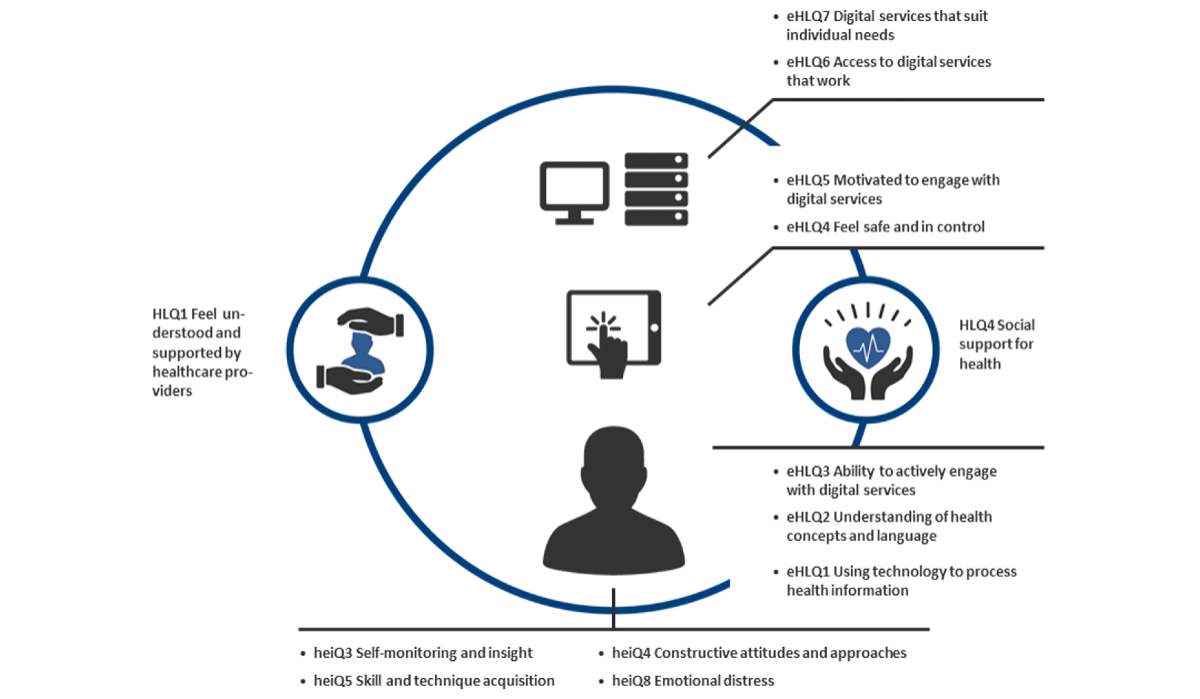
The 13 dimensions of the READHY (reproduced from [16], which is published under Creative Commons Attribution 4.0 International License [20]). The 7 eHLQ dimensions describe users’ attributes; the intersection between users and technologies; and users’ experience of systems. The 4 HLQ dimensions add knowledge about the individuals’ capabilities to handle their condition and emotional response. The 2 eHLQ dimensions add knowledge about individuals’ social context (represented by the circle encompassing the individual and the individual’s attributes). eHLQ: eHealth Literacy Questionnaire; heiQ: Health Education Impact Questionnaire; HLQ: Health Literacy Questionnaire; READHY: Readiness and Enablement Index for Health Technology.

Self-rated health was assessed using a single item from the 36-item Short Form Health Survey [21]. The response options ranged from “very bad” to “very good,” graded on a scale from 1 to 5, with values of 1 to 3 indicating low self-reported health and values of 4 to 5 indicating high self-reported health. Age was recorded in years, and sex was categorized as male or female. The response options for educational level were reported based on the International Classification of Education [22]. The 5 levels were “workers education” (eg, waiter), “skilled in craftsmanship,” “short-cycle higher education,” “medium-cycle higher education,” and “longer education.” Low educational level was categorized as scores of 1-3 and high educational level was categorized as scores of 4-5.

### Data Analysis

Data were presented as mean (SD) for continuous variables and numbers (proportions) for frequencies. Pearson product-moment correlation *r* was used to examine the correlation between self-rated health and READHY values. The degree of the correlation was defined by the *r* value, with 0.10 to 0.29 being weak, 0.30 to 0.49 being moderate, and 0.50 to 1.00 being a strong correlation [23]. Welch 2-sample *t* test (2-tailed) was used to compare READHY scores between recipients with primary and secondary prophylactic ICD indication.

### Cluster Analysis

Individuals were divided into profiles using k-means cluster analysis based on their READHY scores. The objective of the cluster analysis was to identify a profile characterized by particularly low response values across all READHY dimensions. Given the consistently low response values, this group was considered to be of particular clinical relevance for examination and comparison with profiles displaying higher response values.

Performing a k-means cluster analysis requires a prespecification of the number of clusters before the analysis can be conducted. K-means cluster analysis with 3, 4, and 5 clusters were tested in 10 iterations to determine which number of clusters had the most clinically relevant distribution. The seed value of this distribution was then saved, so that all future calculations were made from the same distribution.

Differences among the identified profiles concerning their sociodemographic characteristics and ICD indication were assessed using the Fisher exact test for categorical variables and one-way ANOVA for continuous variables. The results of the one-way ANOVA were presented with *P* values, effect size was calculated as eta-square (η²), and Tukey multiple comparisons of means were used to assess which groups means differed significantly from each other.

Statistical calculations were performed using R (version 1.4.1717; R Core Team).

### Explanatory Interviews

This section is reported according to COREQ (Consolidated Criteria for Reporting Qualitative Research) checklist [24]. Individual semistructured interviews were conducted with 6 participants recruited as described above. All interviews were conducted in person, at a location selected by the participant (home, hospital, or university). The interviews were led by the first author, NR (female), who had no previous relationship with the participants. Each interview began with a thorough introduction to the project including the purpose of interviewing and the professional background of the interviewer. Furthermore, participants were informed that the interview was being recorded for the purpose of transcribing the conversation for further analysis. In this context, the elements of the consent form and information sheet were reviewed with the participant. Present at the interviews were the participant and the 2 first authors, NR and DB. Field notes were made during the interview by DB. The interviewer, NR, holding a master’s degree in health informatics from the University of Copenhagen, is trained in conducting qualitative analyses. In addition, throughout the entire research period, the interviewer received continuous supervision from experienced researchers within the author group, LK and MKW.

A guide for the semistructured interviews was developed based on the READHY framework (Multimedia Appendix 1). The intention of the interviews was to explore the participant’s perspectives on becoming a recipient of an ICD. The interview duration varied from 30 to 60 minutes, with a mean duration of 44.5 (SD 10.81) minutes. Interviews were conducted at various locations, including the hospital (n=2), the patients’ homes (n=3), and at the university (n=1), accommodating the preferences of the individual participants.

Following the conclusion of each interview, a verbatim transcription was meticulously generated from the digital audio recordings. This transcription process ensured that data were accurately and comprehensively captured for subsequent analysis. The analysis of the interview data was carried out using a content analysis with an abductive approach [25]. The software package NVivo12 (Lumivero) was used. The coding was based on the READHY framework with the main categories: self-management (6 notes), social support (4 notes), and digital health literacy (4 notes). Participants have not been presented with the transcribed data nor provided feedback on the findings.

### Ethical Considerations

This study adheres to the ethical principles outlined in the Declaration of Helsinki [26]. The Danish Data Protection Agency approved the handling of data under journal P-2019-78, I-Suite 6423. Furthermore, permission to conduct the study was obtained from the heads of the Department of Cardiology at Rigshospitalet. All participants provided individual written informed consent before completing the questionnaire and participating in the interviews. Participants were informed of the voluntary nature of their participation, their right to withdraw at any time, and how their data would be used for research purposes.

According to section 14(2) of the Danish Act on Committees, health science questionnaire surveys and interview studies that do not involve human biological material do not require reporting or approval from the Danish National Centre for Ethics. Due to this exception, there were no approvals required.

All data collected were anonymized to ensure confidentiality. Personal identifiers were removed, and all data were stored securely in compliance with General Data Protection Regulation and institutional data protection regulations. The data were only accessible to the research team, ensuring the participants’ privacy was maintained.

No compensation was provided to participants for their involvement in this study. However, participants were made aware that their participation would contribute to advancing knowledge in ICD rehabilitation and the potential implementation of digital tools in the rehabilitation process.

## Results

### Overview

In total, 79 participants were included in this study. The participating recipients had a total of 29 primary and 47 secondary prophylactic indications. In 3 participants, the device indication was unknown.

### Sociodemographic Characteristics

The mean age of the 79 participants who completed the survey was 60.4 (SD 12.3) years. The distribution was 73% (56/77) male, and 63% (49/78) had a secondary prophylactic ICD indication. The participants originated from the Capital Region of Denmark and the region of Zealand, Denmark.

### Comparison of READHY Scores and Prophylactic ICD Indication

A comparison of READHY scores of those with primary and secondary prophylactic ICD indications is shown in Table 1. Lower READHY scores were observed for all 13 READHY dimensions for those with primary prophylactic indications compared to those with secondary prophylactic indications, which were significant for HQL1 (*P*=.01), HLQ4 (*P*<.001), eHLQ2 (*P*=.03), eHLQ4 (*P*<.001), and eHLQ6 (*P*=.05).

**Table 1:** Comparison of READHY^a^ scores of recipients with primary and secondary prophylactic ICD^b^ indication (N=76).

READHY dimensions	*P* value	Primary prophylactic indication	Secondary prophylactic indication
heiQ^c^3: Self-monitoring and insight	.46	2.95	3.02
heiQ4: Constructive Attitudes and Approaches	.09	3.01	3.14
heiQ5: Skill and Technique Acquisition	.97	2.85	2.95
heiQ8: Emotional Distress (reversed scale)	.98	2.77	2.95
HLQ^d^1: Feeling understood and supported by healthcare providers	.01	3.03	3.23
HLQ4: Social support for health	<.001	2.89	3.46
eHLQ^e^1: Using technology to process health information	.69	2.81	2.99
eHLQ2: Understanding of health concepts and language	.03	3.01	3.17
eHLQ3: Ability to actively engage with digital services	.22	2.96	3.09
eHLQ4: Feel safe and in control	<.001	3.13	3.31
eHLQ5: Motivated to engage with digital services	.14	2.88	3.1
eHLQ6: Access to digital services that work	.05	2.99	3.16
eHLQ7: Digital services that suit individual needs	.77	2.79	2.98

^a^READHY: Readiness for Health Technology Index.

^b^ICD: implantable cardioverter defibrillator.

^c^heiQ: Health Education Impact Questionnaire.

^d^HLQ: Health Literacy Questionnaire.

^e^eHLQ: eHealth Literacy Questionnaire.

### READHY for Health Technology

Table 2 displays 4 health technology readiness profiles, organized in ascending order based on their average READHY scores. Profile 3 consistently exhibited sufficiency across all scales, while profile 2 was not only lower than profile 3 mostly in eHealth dimensions but also showed a sufficient level across all scales. Profile 1 showed a sufficient level on scales related to self-management and support, but insufficient levels on 5 eHealth Literacy scales except on eHLQ4 and eHLQ2. Profile 4 showed a generally insufficient level across the scales, except on HLQ1, eHLQ2, eHLQ4, and eHLQ5.

**Table 2:** Four health technology readiness profiles on the READHY^a^ scale ranged from 1 (Strongly disagree) to 4 (Strongly agree; N=79). Profiles are listed from the lowest average score (left) to the highest scores (right)—highlighting the difference between each profile.

READHY dimensions			Profiles						
			4 (n=12)		1 (n=9)		2 (n=32)		3 (n=26)
**Self-management, mean score**									
	hei^b^Q3 (Self-monitoring and insight)	2.69		3.04		2.87		3.26	
	heiQ4 (Constructive Attitudes and Approaches)	2.35		3.16		2.93		3.65	
	heiQ5 (Skill and Technique Acquisition)	2.21		2.97		2.81		3.36	
	heiQ8 (Emotional Distress; reversed)	1.80		3.56		2.80		3.35	
**Support, mean score**									
	HLQ^c^1 (Feeling understood and supported by healthcare providers)	2.77		3.17		2.97		3.55	
	HLQ4 (Social support for health)	2.58		3.13		3.19		3.68	
**eHealth literacy, mean score**									
	eHLQ^d^1 (Using technology to process health information)	2.67		2.31		2.76		3.51	
	eHLQ2 (Understanding of health concepts and language)	2.82		2.84		2.93		3.58	
	eHLQ3 (Ability to actively engage with digital services)	2.60		2.35		2.93		3.67	
	eHLQ4 (Feel safe and in control)	3.08		2.87		3.06		3.73	

^a^READHY: Readiness for Health Technology Index.

^b^heiQ: Health Education Impact Questionnaire.

^c^HLQ: Health Literacy Questionnaire.

^d^eHLQ: eHealth Literacy Questionnaire.

### Characteristics of Profiles

Differences in sociodemographic characteristics between profiles are presented in Table 3. A difference in age (*F*_3,70_=3.1, *P*=.03, η²=0.12) was observed. The biggest difference in age was observed between profile 4 and profile 3 (*P*=.03) and between profile 4 and profile 1 (*P*=.07). A difference in self-rated health (*F*_3,75_=6.4, *P*=.001, η²=0.20) was observed between the 4 profiles. The biggest difference in self-rated health was observed between profile 4 and profile 3 (*P*<.001) and between profile 3 and profile 2 (*P*=.01). No difference in sex and educational level was found. When examining for differences between the profiles with respect to ICD indication, no significant differences were found (*P*=.62). However, the percentage receiving the ICD on primary prophylactic indication in the “low-level group” was 50% (6/12) compared with the “high-level group” with only 23% (6/26). Self-rated health and level of education are measured and presented as described in the methods.

**Table 3:** Sociodemographic characteristics of participants (N=79) across profiles. Data are presented as mean (SD) for continuous variables and numbers (proportions) for frequencies.

Characteristics		All (N=79)	Profile 1 (n=9, 11%)		Profile 2 (n=32, 40%)		Profile 3 (n=26, 33%)		Profile 4 (n=12, 15%)		*P* value		
**Gender, n (%)**												.45	
	Women	21 (27)	1 (11)		8 (25)		9 (35)		3 (25)				
	Men	56 (71)	8 (89)		24 (75)		15 (58)		9 (75)				
	Unknown sex	2 (2)	0 (0)		0 (0)		2 (8)		0 (0)				
**Age (years), mean (SD)**		60.38 (12.3)	66 (10.0)		63 (12.7)		58 (12.8)		53 (7.8)		.03		
**Highest attained level of education, n (%)**													.27
	Long education	29 (37)	4 (44)		10 (31)		12 (46)		3 (25)				
	Short education	40 (51)	4 (44)		20 (62)		11 (42)		5 (42)				
	Unknown education	10 (13)	1 (11)		2 (6)		3 (12)		4 (33)				
**Self-rated health, n (%)**													.001
	High self-rated health	43 (54)	6 (67)		15 (47)		20 (77)		2 (17)				
	Low self-rated health	36 (46)	3 (33)		17 (53)		6 (23)		10 (83)				
**Prophylactic indication, n (%)**													.62
	Primary	29 (37)	4 (44)		13 (41)		6 (23)		6 (50)				
	Secondary	47 (60)	5 (56)		18 (56)		18 (69)		6 (50)				
	Unknown	3 (4)	0 (0)		1 (3)		2 (8)		0 (0)				

### Interview Findings

To explore how differences in READHY scores related to the participants’ experiences of becoming recipients of an ICD, we conducted interviews with representatives from profile 3 and profile 4. Profile 4, characterized by the lowest scores in 12 out of 13 READHY scales and lowest self-rated health, was contrasted with profile 3, which demonstrated the highest scores in all 13 scales as well as self-rated health. For the interviews, we recruited 3 participants from profile 3, here on after referred to as the “high-level group,” and 3 participants from profile 4, here on after referred to as the “low-level group.” These interviews revealed significant differences in how individuals from these groups were able to manage their condition, perceived the support they received, and approached digital proficiency.

### Self-Management

All participants engaged in self-management practices addressing their physical and mental well-being. However, there was a distinction in how self-management was interpreted within the “high-level group” compared to the “low-level group.” Participants belonging to the “high-level group” described their pre-ICD implantation lifestyle as characterized by daily physical exertion, which they expressed a strong desire to sustain. For instance, P3 stated:

I used to bike to work throughout the year, covering approximately 10 kilometers each way. I engaged in workouts at least twice a week and participated in a weekly spinning class. Exercise, to me, equates to an enhanced quality of life, both presently and prior to my illness. At present, I attend one or two spinning classes weekly, which I prefer not to disclose to my doctors, as they disapprove.

In contrast, no one in the “low-level group” used physical activity as a means to preserve their health.

Participants belonging to the “low-level group” approached self-management in a distinct manner, which primarily involved adhering to medical advice regarding medication adherence and health care appointments, particularly evident when asked about their self-care practices. For example, P2 and P5 articulated:

After doctors’ appointments I am more sensitive and attentive to my body. Naturally, the plan is to initiate lifestyle changes, which I have gradually commenced.“ And ”It seems like that's all I'm engaged in - devoting my time to managing my health. I visit the hospital constantly, and I mean incessantly. Furthermore, I was enrolled in a heart rehabilitation program last year.

For individuals within the “low-level group,” a recurring subject was found, wherein the participants lived with constant awareness and apprehension regarding their condition. For instance, when asked, “During your daily routine, when do you find yourself contemplating your ICD?” P1 articulated “Constantly! It occupies my thoughts incessantly.” P2 concurred, stating:

I think about it every time I shower, change my clothing, and when I retire for the night; those are the moments when it preoccupies my mind the most. Additionally, I grapple with mental concerns such as whether it would effectively function in the event of an unforeseen circumstance.

Similarly, P5 shared, “All the time! I am in a constant state of unease.”

When the same question was asked to participants belonging to the “high-level group,” the responses conveyed a sense of calm and trusting emotional state. As exemplified by P4 and P6:

My perspective has been somewhat matter of fact; I needed to have this device implanted, and that is simply the way it is. Beyond that, I have not dwelled on it extensively.

P4

After a full day at work, I may experience some soreness, but it reminds me of how reassuring it is to have it watching over me.

P6

### Support

#### Social Support

In the management of their ICD, participants who felt a lack of social support from family and friends during the rehabilitation process have heightened emotional distress, necessitating additional support from health care professionals. Without substantial social support from family and friends, the perception of support from health care professionals during their hospitalization and rehabilitation process became crucial. A lack of social support affected the participant’s ability to place trust in the ICD technology and their capacity to adapt calmly to life with an ICD.

The significance of having access to supportive relatives or spouses was emphasized by the contrast in how the 2 groups used and derived comfort from sharing their concerns with close family members. The “high-level group” experienced tremendous comfort in doing so, whereas the “low-level group” tended to conceal their feelings and kept their worries to themselves. For instance, P4 remarked:

Discussing things with my family and my wife, who was present at the time of my cardiac arrest, and having those conversations with people who asked about my experiences, has actually proven more beneficial than speaking with the psychologist.

This contrasted with the experiences of recipients in the “low-level group,” who perceived their condition as more burdensome for their families than as a source of support. P2 explained:

You may want to confide in your family, but not be completely honest about how frightened you have been and still are about the future. It's a delicate topic. My family was deeply shaken, and they may not wish to revisit it.

Similar sentiments were expressed by P5:

My children are 22 and 23 years old, but they have been extremely anxious. Being a single mom and trying to stay strong for them is challenging. Yet, they want me to share my feelings. It's just very tough at times.

#### Professional Support

Participants who lived alone exhibited a greater demand for support and information from health care professionals when compared with participants living with a spouse. Those living alone consistently expressed dissatisfaction with the support provided by health care professionals and commonly expressed high levels of emotional distress, as well as a lack of information, support, and therapeutic options. P1 felt that his needs were overlooked and emphasized the need for more information about his condition, stating:

When you get admitted here, you receive absolutely no information. None. That is a flaw. I was operated on at 2 a.m., and by 9 a.m., I was approached by a professor and a nurse who wanted to recruit me for a study. That was bewildering. After surgery, your mind is in turmoil, and here they are asking me to participate in a study.

In addition, another participant who was living alone, P5, expressed dissatisfaction with the lack of fulfillment and comprehension of her needs during her hospitalization, particularly concerning the therapy options offered after surgery. She stated:

During my hospitalization, I attended a few sessions with their psychologist, but it didn't resonate with me at the time. They advised me to go for forest walks and visit the library to socialize. That wasn't what I needed.

In contrast, all participants living with a partner consistently reported the support provided by health care professionals as highly satisfactory. P4 stated:

I felt safe from the moment I woke up in the hospital and throughout my entire stay. I have been extremely pleased with the care and treatment I received here.

P6 similarly expressed positive impressions, saying:

I wish I could write an article about it; it felt like a five-star hotel. They treated me like royalty, providing me with detailed information, time, and care. We were deeply impressed by the dedication and attention they gave us.

#### Digital Health Literacy

Participants from both the “high-level” and “low-level” groups expressed a consistent readiness and ability to engage with digital health care services and use various technological tools as part of their recovery process. They shared a common inclination for monitoring their health data, seeking health information online, and accessing personal health records through digital platforms. There was no noticeable difference in motivation for digital rehabilitation between the 2 groups, potentially due to their recruitment from a rehabilitation program rather than during hospitalization. Moreover, both groups displayed similar engagement with other health-related technologies, such as smartwatches and pulse oximeters, indicating their willingness to embrace technology for a digitalized rehabilitation experience tailored to their needs.

A participant belonging to the “low-level group,” P5, detailed her utilization of various technologies for managing her condition:

I have been using my Apple Watch since I received my first pacemaker. Sometimes, I would feel unwell and worry about my pulse being too low. Tracking it on my watch gives me peace of mind. Additionally, I regularly log in to my online electronic health record to stay informed about any updates. The more information I acquire, the more at ease I feel.

Similarly, P4 belonging to the “high-level group” expressed:

I purchased an actual pulse oximeter when my condition first arose. I told my wife that I needed one. I have an imperative need to comprehend what is transpiring.

#### ICD Indication

One distinguishing characteristic of recipients within the “low-level group” was their lack of trust in the ICD technology and the high levels of emotional distress they experienced living with an ICD. It is noteworthy that the 3 recipients belonging to the “low-level group” had previously been diagnosed with heart-related conditions before receiving the ICD, which contrasts with the participants belonging to the “high-level group” who had no such previous diagnoses. The recipients with an ICD who have primary prophylactic indication consistently exhibit notably low READHY scores, especially in the domain of social support, when compared to recipients with secondary prophylactic indication. Interviews show that the overall health status of the recipient before ICD placement is an essential determinant influencing the patient’s ability to manage the condition. Importantly, the interviewer had no previous knowledge of which group the interviewed participants belonged to.

#### Patient Vignettes

Based on data presented in Tables 2 and 3 and the qualitative interviews, we have created 2 patient vignettes, which are presented below. These demonstrate how the text vignettes can make the profiles more vivid for health care professionals.

##### Vignette for the Low-Level Group

This is a male individual aged 53 years with low physical activity levels and low self-rated health, diagnosed with other comorbidities before ICD implantation. The patient is unmarried, lives alone, has a limited social network, and experiences significant emotional distress due to his condition on a daily basis. He uses health technologies and actively seeks information about his condition online. The “low-level group” of patient requires a high level of support from health care professionals during hospitalization and through their rehabilitation process.

##### Vignette for the High-Level Group

This is a male individual aged 58 years with a high level of physical activity and high self-rated health, who maintains good health and has no comorbidities before his ICD implantation. The patient cohabits with a partner and has an extensive social network. He maintains a positive attitude toward his condition and incorporates health technologies into his daily routine.

## Discussion

### Principal Findings

The purpose of this study was to demonstrate how profiles and patient vignettes can be developed using the READHY instrument to make health care professionals aware of differences in patient’s needs, resources, and capabilities in relation to their health technology readiness, including their emotional state. Using cluster analysis, 4 clinically relevant profiles were developed. The most distinct profiles we found were profile 3, characterized by highly sufficient READHY scores across all dimensions, and profile 4, characterized by 9 insufficient READHY scores (below 2.7), displaying only slight sufficiency within digital literacy. Sociodemographic characteristics, age, and self-reported health differed among the profiles, with the youngest patients having the lowest READHY scores. No significant differences were found in sex, level of education, or ICD indication. This underpins the need other than these classical characteristics to inform the health care professionals to understand their patients. The interviews provided valuable insights into the perspectives of the profiles, emphasizing the crucial role of social support, particularly for those living alone, who required more professional support. These insights were particularly relevant with regard to emotional distress and perceived support levels from family and health care professionals.

Individuals with no or a short history of poor health conditions tended to adapt more positively to life post-ICD implantation, compared with those with a longer history of poor health conditions. This suggests that it may be significant to take the patient’s previous and current status of health into consideration in the treatment of them. Interestingly, interviewees belonging to both the low and high-level groups embraced technology to a high extent, signifying that in recipients of an ICD, physical health is not related to the usage of technology.

### Profile Characteristics

#### Age and Self-Rated Health

We found significant differences in age and self-reported health among the recipients of an ICD in different profiles, but no significant difference in sex, educational level, or ICD indication. Profile 4, which represents individuals with the lowest READHY scores, is comprised of individuals who are, on average, 13 years younger than those in the oldest profile. This contrasts with previous research, where older adults tended to have poorer health outcomes [15]. The youngest patients had the lowest scores in self-rated health, indicating that age alone may not be a strong predictor of ICD-related health outcomes. This suggests the importance of considering other factors such as other long-term health conditions and self-rated health status when assessing patient needs, resources, and capabilities, rather than age.

#### Social Support

In alignment with previous findings [15], our interview data show that emotional and social support from a partner or spouse plays a role in addressing emotional concerns after ICD placement. The participants living with a spouse reported an exceptionally high level of received care from health care professionals and had little need to seek additional support. Conversely, participants living alone expressed feelings of abandonment, lack of information, and insufficient care from health care professionals.

The impact of social support on mental well-being is further evident in the difference in emotional concerns between the “high-level” and “low-level” groups. The “high-level group” expressed trust in their ICD and had fewer daily worries about their condition, whereas all participants in the “low-level group” reported doubts about their ICD’s effectiveness and ongoing concerns about their future health. Therefore, the presence or absence of social support in the form of a spouse or near family is a crucial factor to consider when identifying patients who may require additional support and tailored rehabilitation services.

#### Digital Health Literacy

The recipients of an ICD had relatively high levels of digital health literacy scores in both the “low-level” and “high-level” groups compared to patients with inflammatory bowel disease [14]. The sufficiency of digital health literacy was further confirmed during interviews, where all participants reported regular use of digital health tools in their daily lives. This contrasts with previous research, which suggests limited technology engagement among individuals with chronic illnesses [14]. In our study, recipients of an ICD from various profiles actively embraced technology for health monitoring; sought health-related information online; and used devices such as smartwatches, fitness trackers, and advanced pulse oximeters, regardless of their profile. This collective engagement suggests an opportunity among recipients of an ICD to adopt new digital services and technology.

Our interviews involved individuals from profiles 4 and 3. Profiles 4 and 3 were selected due to having the overall lowest and highest READHY scores, respectively, but it should be noticed that the lowest levels of digital health literacy were found in profile 1.

The characteristics of participants belonging to profiles 1 and 2 should also be considered when planning rehabilitation. Identifying individuals within these intermediate profiles is essential, as they may also exhibit low values in specific dimensions. Profile 1 had a sufficient level within the areas of self-management and social support but was found with lower levels in digital health literacy compared with the other profiles. The introduction of digital technologies may pose a barrier for this group, as they do not possess the same high levels of digital literacy as the other groups. In essence, while they excel in traditional health-related knowledge, they may struggle when it comes to using digital health tools and resources. This group should be approached recognizing their nondigital competence and with a careful introduction of digital solutions.

Profile 2 was the largest group, characterized by having sufficient levels on all scales. Despite having lower levels than those in profile 3, they are considered capable of actively participating in their rehabilitation including complementary digital services and technologies. The key here is to recognize individuals who are less capable than those in profile 4 but still require increased assistance and rehabilitation services, especially within the self-management area.

Due to the fact that recipients of an ICD can be clustered into diverse patient profiles where some have low digital literacy, we advocate retaining the in-person ICD rehabilitation meeting as an available option, particularly for individuals belonging to profiles 1 and 4. This group may benefit from additional support, counseling, and information throughout their recovery process, ensuring a more comprehensive and personalized approach to their care. The interviews indicated that all individuals, regardless of which of the 2 profiles they belonged to, regularly used digital services and found them to be comfortable and reassuring. This suggests that most recipients of an ICD, including those with lower levels of digital health literacy, can benefit from the enhanced integration of technology into the ICD rehabilitation program. Using the READHY instrument to identify profiles and their associated individuals will serve as a valuable tool in tailoring future ICD treatments to meet individual needs.

#### ICD Indication

Regarding the differences in prophylactic indication, it is important to recognize that the current treatment pathways vary based on the indication. Patients undergoing secondary ICD placement, often due to acute conditions like cardiac arrest, experience a more prolonged hospital stay compared with those undergoing planned, elective, primary ICD placement. Conducting a study that combines both primary and secondary indications for ICD placement involves including a group of patients who have not undergone the exact same treatment process. Despite this, our qualitative analysis remained impartial, as all interviewed participants underwent secondary ICD placement, ensuring a one-to-one basis for comparison.

Recipients with primary ICD indications had lower, but sufficient, levels of all 13 READHY scales compared with those with secondary indications. This was significant in relation to support from both professionals (HLQ1) and relatives or peers (HLQ4); it was also significant in relation to the 3 digital health scales concerning having access to digital services for those who need them (eHLQ6), trusting how their data are handled (eHLQ4), and understanding the health language (eHLQ2). The higher READHY scores from recipients with a secondary indication for ICD placement could be due to their prolonged hospitalization, which gave them more extensive interaction with health care professionals. Another explanation could be that this group has not experienced a prolonged history of poor health, resulting in fewer interactions with the health care sector and potentially fostering a more optimistic outlook.

#### Patient Vignettes

A way to make the profiles more present and recognizable by health care professionals is to create vignettes that describe a particular average person belonging to a specific profile.

The vignettes offer insights into the unique needs, challenges, and behaviors of individuals within the “low-level” and “high-level” groups of this study. By delving into the details of these vignettes, we aim to provide a deeper understanding of how various factors, including health status, social support, and lifestyle, influence the experiences of recipients of an ICD. The vignettes serve as representative examples with the purpose of assisting health care professionals in identifying patient characteristics, ultimately enabling the delivery of more tailored support and care to the population of recipients of an ICD. It remains to be tested in a clinical setting to what extent these vignettes can help the health care professionals in their everyday work.

### Strengths and Limitations

A strength of the study lies in its foundation on an established model previously used in patients with other chronic conditions. The data help translate the understanding of health technology readiness into a new clinical area, providing a fresh perspective for health care professionals in cardiology. This enables them to better meet patients’ needs while considering their resources and capabilities in a digital context, including mental and social aspects.

However, a limitation of this study is the absence of interviews with individuals from profile 1, which is characterized by the lowest level of digital health literacy, particularly in scales eHLQ1, eHLQ3, eHLQ5, and eHLQ7. Including interviews from this group could have yielded valuable insights into the factors contributing to their low digital competence. By not doing so, the depth and comprehensiveness of the data were somewhat limited.

In addition to the above, another potential limitation is the relatively low number of participants, which may introduce a risk of bias, as only those with a high level of self-management ability may have participated. This could also increase the risk of a type 2 error, potentially overlooking differences between profiles in sociodemographic characteristics and self-reported health.

Furthermore, the survey sampling took place over a period of 2 years and 7 months, during which the COVID-19 pandemic occurred, limiting the number of participants that could be included. A multicenter study would have been necessary to achieve a larger sample size within this timeframe. Nevertheless, despite this limitation, the data still contribute significantly to our understanding of recipients of an ICD and the dynamics of their competencies.

Finally, a limitation in interpreting the differences between primary and secondary indications for ICD placement is worth noting. Some individuals in the secondary group may have had preexisting heart conditions, making them more similar to patients in the primary group. Unfortunately, this factor was not accounted for in the study design, as the health care professionals involved no longer had responsibility for these patients. Although differences in READHY scales and self-rated health between the groups suggest this may have been a minor issue, future studies should emphasize assessing preexisting heart conditions and the need for cardiac resynchronization therapy.

### Conclusion

The profiles developed in this study offer a practical tool to translate complex data into a more accessible format, enabling health care professionals to identify individuals who require additional support and those who may benefit from increased online contact. These profiles can be transformed into patient vignettes, presented in a concise text format, which help clinicians recognize specific needs related to self-management, digital health literacy, and experienced support in the context of ICD rehabilitation.

For example, profile 3 demonstrated high readiness scores across all dimensions, indicating strong self-management capabilities and a potential for greater engagement with digital health tools. In contrast, profile 4 had low scores across multiple areas, representing individuals with significant challenges in managing their condition and engaging in a rehabilitation process. These profiles highlight the spectrum of readiness and the need for tailored interventions.

It is equally important to acknowledge intermediate profiles, such as profiles 1 and 2, which exhibit unique needs that demand tailored rehabilitation approaches, particularly in the context of digital health literacy. By understanding the diversity within this population and considering the impact of sociodemographic factors, health status, and social support, health care professionals can provide more personalized and effective care to recipients of an ICD in the future.

## Appendix

Multimedia Appendix 1 Interview guide.

## Abbreviations

COREQ: Consolidated Criteria for Reporting Qualitative Research
eHLQ: eHealth Literacy Questionnaire
heiQ: Health Education Impact Questionnaire
HLQ: Health Literacy Questionnaire
ICD: implantable cardioverter defibrillator
READHY: Readiness for Health Technology Index

## References

[1] Nisam S, Reddy S (2015). The story of … a lead. Europace.

[2] Sommer A, Witt CT, Diederichsen S (2024). Implanterbar cardioverter defibrillator (ICD) [Article in Danish]. Dansk Cardiologisk Selskab.

[3] Al-Khatib SM, Stevenson WG, Ackerman MJ, Bryant WJ, Callans DJ, Curtis AB, Deal BJ, Dickfeld T, Field ME, Fonarow GC, Gillis AM, Granger CB, Hammill SC, Hlatky MA, Joglar JA, Kay GN, Matlock DD, Myerburg RJ, Page RL (2018). 2017 AHA/ACC/HRS guideline for management of patients with ventricular arrhythmias and the prevention of sudden cardiac death: executive summary: a report of the American college of cardiology/american heart association task force on clinical practice guidelines and the heart rhythm society. J Am Coll Cardiol.

[4] Alhakak A, Østergaard L, Butt JH, Vinther M, Philbert BT, Jacobsen PK, Yafasova A, Torp-Pedersen C, Køber L, Fosbøl EL, Mogensen UM, Weeke PE (2022). Cause-specific death and risk factors of 1-year mortality after implantable cardioverter-defibrillator implantation: a nationwide study. Eur Heart J Qual Care Clin Outcomes.

[5] Dunbar SB, Dougherty CM, Sears SF, Carroll DL, Goldstein NE, Mark DB, McDaniel G, Pressler SJ, Schron E, Wang P, Zeigler VL (2012). Educational and psychological interventions to improve outcomes for recipients of implantable cardioverter defibrillators and their families: a scientific statement from the American heart association. Circulation.

[6] Magyar-Russell G, Thombs BD, Cai JX, Baveja T, Kuhl EA, Singh PP, Montenegro Braga Barroso M, Arthurs E, Roseman M, Amin N, Marine JE, Ziegelstein RC (2011). The prevalence of anxiety and depression in adults with implantable cardioverter defibrillators: a systematic review. J Psychosom Res.

[7] Berg SK, Pedersen PU, Zwisler A, Winkel P, Gluud C, Pedersen BD, Svendsen JH (2015). Comprehensive cardiac rehabilitation improves outcome for patients with implantable cardioverter defibrillator. Findings from the COPE-ICD randomised clinical trial. Eur J Cardiovasc Nurs.

[8] Risom SS National klinisk retningslinje for rehabilitering til patienter med atrieflimren, atrieflagren, patienter med endokarditis og patienter behandlet med en implanterbar cardioverter defibrillator (ICD) [Article in Danish]. Danske Fysioterapeuter.

[9] Palshof MK, Jeppesen FKH, Thuesen AD, Holm CS, Brøndum E, Kayser L (2023). Comparison of the level of eHealth literacy between patients with COPD and registered nurses with interest in pulmonary diseases. Computer Methods and Programs in Biomedicine Update.

[10] Krag T, Jørgensen EH, Phanareth K, Kayser L (2023). Experiences with in-person and virtual health care services for people with chronic obstructive pulmonary disease:qualitative study. JMIR Rehabil Assist Technol.

[11] Poulsen HS, Eiriksson SD, Christiansen ASJ, Wingstrand A (2022). Sundhedsprofil 2021 for Region Sjælland og kommuner: »Hvordan har du det?«.

[12] Hallas CN, Burke JL, White DG, Connelly DT (2010). A prospective 1-year study of changes in neuropsychological functioning after implantable cardioverter-defibrillator surgery. Circ Arrhythm Electrophysiol.

[13] Kramer DB, Habtemariam D, Adjei-Poku Y, Samuel M, Engorn D, Reynolds MR, Mitchell SL (2017). The ecisions, nterventions, and oals in implatable cardioverter-defbrillator herap (DIGNITY) pilot study. J Am Heart Assoc.

[14] Nielsen AS, Hanna L, Larsen BF, Appel CW, Osborne RH, Kayser L (2022). Readiness, acceptance and use of digital patient reported outcome in an outpatient clinic. Health Informatics J.

[15] Thorsen IK, Rossen S, Glümer C, Midtgaard J, Ried-Larsen M, Kayser L (2020). Health technology readiness profiles among danish individuals with type 2 diabetes: cross-sectional study. J Med Internet Res.

[16] Kayser L, Rossen S, Karnoe A, Elsworth G, Vibe-Petersen J, Christensen JF, Ried-Larsen M, Osborne RH (2019). Development of the multidimensional Readiness and Enablement Index for Health Technology (READHY) tool to measure individuals' health technology readiness: initial testing in a cancer rehabilitation setting. J Med Internet Res.

[17] Osborne RH, Elsworth GR, Whitfield K (2007). The Health Education Impact Questionnaire (heiQ): an outcomes and evaluation measure for patient education and self-management interventions for people with chronic conditions. Patient Educ Couns.

[18] Osborne RH, Batterham RW, Elsworth GR, Hawkins M, Buchbinder R (2013). The grounded psychometric development and initial validation of the Health Literacy Questionnaire (HLQ). BMC Public Health.

[19] Kayser L, Karnoe A, Furstrand D, Batterham R, Christensen KB, Elsworth G, Osborne RH (2018). A multidimensional tool based on the eHealth literacy framework: development and initial validity testing of the eHealth Literacy Questionnaire (eHLQ). J Med Internet Res.

[20] Attribution 4.0 International (CC BY 4.0). Creative Commons.

[21] Ware JE, Sherbourne CD (1992). The MOS 36-item Short-Form Health Survey (SF-36). I. Conceptual framework and item selection. Med Care.

[22] (2017). International Standard Classification of Education (ISCED). UNESCO Institute for Statistics.

[23] Pearson's correlation coefficient. Statistics Solution.

[24] Booth A, Hannes K, Harden A, Noyes JP, Harris J, Tong A (2014). COREQ (Consolidated Criteria for Reporting Qualitative Studies). Guidelines for Reporting Health Research: A User's Manual.

[25] Elo S, Kyngäs H (2008). The qualitative content analysis process. J Adv Nurs.

[26] WMA Declaration of Helsinki - ethical principles for medical research involving human subjects. The World Medical Association.

