# Supplementary material for: Technology Readiness Level and Self-Reported Health in Recipients of an Implantable Cardioverter Defibrillator: Cross-Sectional Study
Source: JMIR Cardio. 2025 Feb 6;9:e58219. doi: 10.2196/58219 (PMC11843055; doi:10.2196/58219)
Supplement: Multimedia Appendix 1 [file cardio_v9i1e58219_app1.docx]

**Multimedia Appendix 1**

**Interview guide**

**Theme: Self-management and emotional distress**

- Where in your everyday life do you think about having an ICD?
- Do you feel that there are things you do differently or avoid doing after receiving your ICD?
- Can you provide some examples of when it affects things you do and plan?
- Is there anything you have to do without?
- Do you feel that you spend a lot of time taking care of your health?
- What helps you in your everyday life?
- What makes you happy in your everyday life?

**Theme: Support**

- What thoughts/considerations have you had during the course of your treatment?
- Have there been any experiences in your treatment that you have reflected on?
- What topics have needed to be discussed during your treatment?
- Have you talked to healthcare professionals or your doctor about your thoughts?
- Have you talked to your family or friends about your thoughts?

**Theme: Digital literacy**

- What technology do you use in your daily life?
- Do you search for information about your health, and if so, where do you look?
- Can you provide some examples of when you search for information?
- What would prompt you to seek information?
